# Isolation and Characterization of Microsatellite Loci in the Asian Rice Gall Midge (*Orseolia oryzae*) (Diptera: Cecidomyiidae)

**DOI:** 10.3390/ijms12010755

**Published:** 2011-01-20

**Authors:** Jagadish S. Bentur, Deepak Kumar Sinha, Padmavathy, Charagonda Revathy, Mayandi Muthulakshmi, Javaregowda Nagaraju

**Affiliations:** 1 Directorate of Rice Research, Rajendranagar, Hyderabad 500030, India; E-Mails: deepak22sinha@yahoo.co.in (D.K.S.); padma_bio@yahoo.com (P.); revathycharagonda@gmail.com (C.R.); 2 Centre for DNA Fingerprinting and Diagnostics, Hyderabad 500001, India; E-Mails: muthulaxmi@cdfd.org.in (M.M.); jnagraju@cdfd.org.in (J.N.)

**Keywords:** rice, biotypes, virulence, Oryza sativa, SSR markers, pest of rice

## Abstract

Microsatellite loci were isolated from the genomic DNA of the Asian rice gall midge, *Orseolia oryzae* (Wood-Mason) using a hybridization capture approach. A total of 90 non-redundant primer pairs, representing unique loci, were designed. These simple sequence repeat (SSR) markers represented di (72%), tri (15.3%), and complex repeats (12.7%). Three biotypes of gall midge (20 individuals for each biotype) were screened using these SSRs. The results revealed that 15 loci were hyper variable and showed polymorphism among different biotypes of this pest. The number of alleles ranged from two to 11 and expected heterozygosity was above 0.5. Inheritance studies with three markers (observed to be polymorphic between sexes) revealed sex linked inheritance of two SSRs (Oosat55 and Oosat59) and autosomal inheritance of one marker (Oosat43). These markers will prove to be a useful tool to devise strategies for integrated pest management and in the study of biotype evolution in this important rice pest.

## 1. Introduction

The Asian rice gall midge, *Orseolia oryzae* (Wood-Mason) (Diptera: Cecidomyiidae), a major pest of rice, *Oryza sativa* L. [1], forms leaf-sheath gall called silver shoot. The maggot, hatching from the egg, crawls down the leaf sheath, feeds on the apical meristem and induces formation of gall, which renders the tiller sterile resulting in grain yield loss. It is the third most economically important pest of rice in India causing an average annual yield loss worth US$ 80 million [2]. Host plant resistance is the most effective, eco-friendly and cost efficient means of managing the pest to alleviate crop loss [3]. Resistance in plant is controlled by a single, generally, dominant gene. To date, 11 resistance genes have been identified [4]. But the resistance in the commercial rice varieties is short lived due to the ability of the insect to rapidly evolve virulent populations, called biotypes. So far, seven distinct biotypes of the pest have been characterized in India [5]. Pest populations, however, are not homogeneous in biotype composition in time and space [6]. In order to understand the process of evolution of biotypes characterization of diverse biotypes and studies on gene flow among populations is essential. Molecular markers have proven invaluable in such studies. Behura *et al*. [7] used SCAR/RAPD markers for biotype distinction. AFLP markers have been used in the study of biodiversity of the gall midge populations from 15 sites across five Asian countries [8]. However, these markers lacked reproducibility or are difficult to use. In our earlier studies [5], we devised methods for differentiating the biotypes and screened for different biotypes, which resulted in establishment of a pure culture of three biotypes. Hence, we attempted to develop reliable and easy to use PCR-based simple sequence repeat (SSR) markers for this insect species. In the current work we have, for the first time, identified simple sequence repeats from the Asian rice gall midge genome. The sequence information from these repeat regions was used to develop 90 SSR markers to reveal polymorphism between the three biotypes and sexes and studied inheritance of three markers.

## 2. Results and Discussion

### 2.1. Microsatellite Loci and Polymorphism

Out of the 1635 recombinant clones screened, 1309 (80%) clones had the insert of desired size (≥500 bp). Among these, 170 (13%) clones had repeats of varying lengths. From these, 90 microsatellite loci were selected and primers were designed using Primer 3 software (http://frodo.wi.mit.edu/cgi-bin/primer3) [9]. Of the 90 loci studied, the majority contained dinucleotide repeat motifs (72%), followed by tri- (15.3%), and more complex (12.7%) repeat motifs. Dinucleotide repeats mostly consisted of GT (17.7%) repeats, followed by GA repeats (12.2%). The higher percentage of dinucleotide repeats found was in contrast to the report of SSRs found in another midge, the Hessian fly [10], which had an abundance of trinucleotide repeats.

After standardization, 15 loci (Table 1) were found to be polymorphic between the biotypes and sexes, whereas 75 loci were found monomorphic (Supplementary Table 1). Number of alleles of these 15 markers ranged from two (Oosat21, Oosat46 and Oosat78) to 11 (Oosat59) per locus. All the loci had expected heterozygosity of more than 0.5 in different biotypes and therefore these markers can be considered as good for parentage analysis. The range of observed and expected heterozygosity was calculated to be 0 to 1 and 0.304 to 0.910, respectively. Polymorphic information content (PIC), a measure of informativeness of a marker, calculated using Cervus v2.0 [11], ranged from 0.305 to 0.877; 14 markers with >0.5 PIC are considered highly informative in terms of their suitability for diversity analysis. Oosat26, Oosat55 and Oosat83 were most informative, while Oosat21 the least, against all the three biotypes. Despite multiple alleles, frequency of alleles varied in different biotypes (Table 2). Allele frequency of the predominat allele for each of the biotypes varied from 0.15 to 0.8. Some markers showed high allele frequency for some alleles, therefore fixation tendency of all markers was analysed. Fixation table (Table 3) was generated with following fixation values; FIS, to measure the deviation of genotypic frequencies from panmictic frequencies in terms of heterozygous deficiency or excess, FST, reduction in heterozygosity in a subpopulation due to genetic drift and FIT, overall inbreeding coefficient of an individual relative to the total population. For three loci (Oosat24, Oosat35 and Oosat46) negative FIS values indicated heterozygote excess (outbreeding). For other locus, positive values indicated heterozygote deficiency (inbreeding). FST values up to 0.05 indicate negligible genetic differentiation within the population which was observed for locus Oosat21, Oosat83 and Oosat88. For all the polymorphic loci, the Ewens-Watterson Test for Neutrality was also performed and it was observed that except for the locus Oosat88, all the loci were found neutral. Oosat88 was not behaving neutral in GMB4M population (at 95% confidence level).

Linkage disequilibrium studies (using Fisher’s method) after sequential Bonferroni correction revealed that Oosat26, Oosat36 and Oosat88 were significantly associated with each other. Also the markers Oosat24 and Oosat46, Oosat35 and Oosat21 were associated with each other, whereas other markers segregated independently in the population. *P*-value (HWE) mentioned in Table 1, was calculated according to Guo and Thompson [12]. Observed deviation from Hardy Weinberg equilibrium for some markers (Oosat3, Oosat21, Oossat35, Oosat43, Oosat79 and Oosat88; *P* > 0.05) may be the result of inbreeding, natural selection or genetic drift. Null alleles were also detected using Microchecker v.2.2.3 [13] for markers Oosat16, Oosat26, Oosat36, and Oosat83, and these could be as a result of mutations occurring in the flanking regions, preventing one or both of the primers from binding. Other polymorphic markers did not show null alleles. Markers without null alleles, give a better estimate of allele frequencies.

BLASTX analysis was performed using the sequence information of the 15 polymorphic loci which revealed that Oosat78 showed homology to tyrosyl-dna phosphodiesterase of *Culex quinquefasciatus* (*E* value: 1 × 10^−10^). The SSR repeat sequence was found to be present in the intron region of the gene. The gene has been reported to be from the phopholipase D family, which includes diverse groups of enzymes involved in phopholipid metabolism, a bacterial toxin, viral envelop proteins, and bacterial nucleases [14]. BLASTN analysis, and also BLASTX analysis with Hessian fly sequences showed insignificant similarity.

### 2.2. Inheritance of SSR Markers

**Figure 1:**
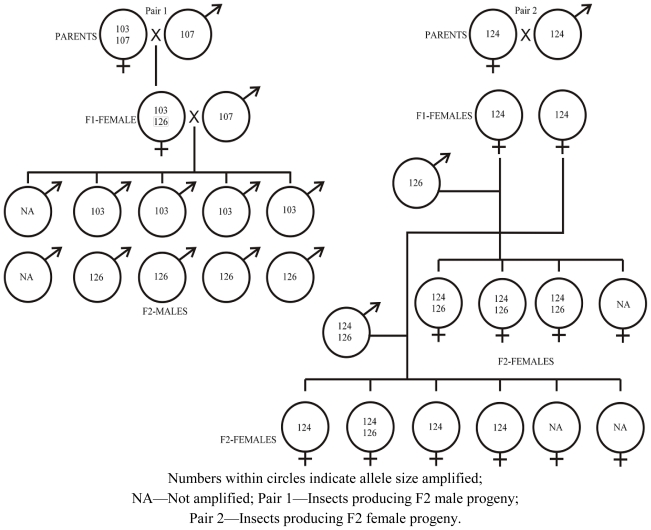
Pedigreed crosses revealing inheritance of Oosat55 in gall midge biotype 4M.

**Figure 2:**
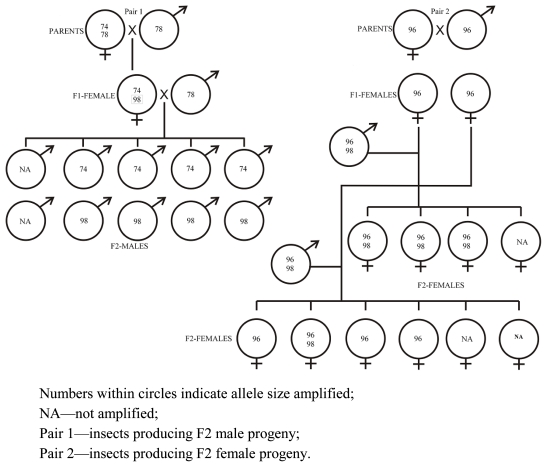
Pedigreed crosses revealing inheritance of Oosat59 in gall midge biotype 4M.

**Figure 3:**
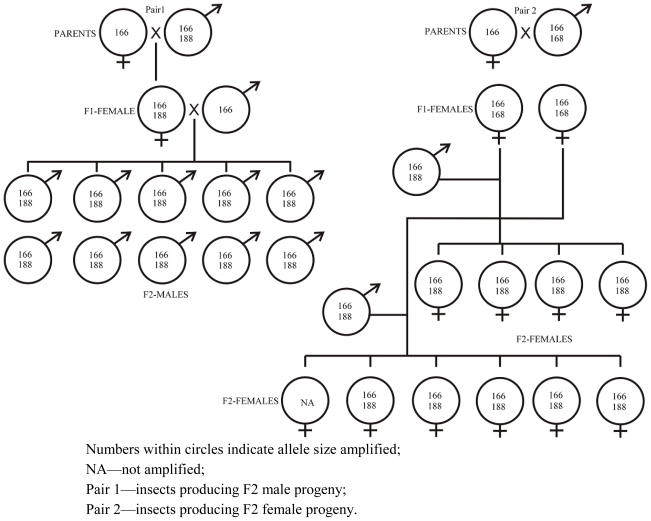
Pedigreed crosses revealing inheritance of Oosat43 in gall midge biotype 4M.

The inheritance pattern of three of the labeled markers, Oosat43, Oosat55 and Oosat59, was studied in F_2_ families of GMB4M through pedigreed crosses. Inheritance of two of the markers—Oosat55 and Oosat59—proved to be sex linked. The male progeny inherited alleles only from the female parent (Figures 1 and 2); but female progeny inherited alleles from both the male and female parents. However, the pattern of inheritance of alleles of Oosat43 suggested an autosomal pattern as the alleles were inherited from both the male and female parents (Figure 3). Inheritance pattern of these markers confirmed existence of sexual dimorphism [15] and the abnormal chromosomal cycle typical of Cecidomyiidae [16,17]. Sex linked markers may be useful in tagging virulence alleles that are often sex linked [18]. Further, studies on inheritance of the markers also suggested some degree of instability with the appearance of novel non-parental alleles appearing in the offspring (Figures 1 and 2). Thus, the present study reports and confirms the usefulness of SSR markers for the rice gall midge.

## 3. Experimental Section

### 3.1. Insect Colonies

Colonies of gall midge biotypes (GMB) GMB1, GMB4 and GMB4M are being maintained in the greenhouse at the Directorate of Rice Research under physical isolation and on appropriate differential rice genotypes [5]. Iso-female families were initiated with two founding pairs, since a single female will produce either all male (androgenic) or all female (gynogenic) progeny. Eight to ten F_1_ pairs were mated separately to obtain F_2_ adults, which were then pooled to initiate the family. At least 10 generations were reared before using these insects for DNA extraction and for inheritance studies.

### 3.2. Isolation of Microsatellite Loci

DNA was extracted from the iso-female families of adult midges. The insects were crushed in an extraction buffer (0.1 M NaCl; 0.1 M Tris-HCl, pH 9.1; 0.05 M EDTA; 0.05% SDS), and extracted once with phenol:chloroform:isoamyl alcohol (25:24:1), and then once with chloroform:isoamyl alcohol (24:1). The purified genomic DNA was ethanol precipitated and resuspended in sterile distilled water after rinsing the pellet in 75% alcohol. The pooled DNA from the three biotypes was used for the purpose of library generation.

The library was constructed using hybridization capture approach of Glenn and Schable [19]. Genomic DNA was digested with the restriction enzymes *Rsa I* and *Xmn I* and ligated with the super SNX double stranded linkers on both sides to provide the primer binding site for subsequent PCR steps. They also provided sites for cloning in the vectors. Dynabead enrichment for Microsatellite containing DNA was performed. From the hybridized DNA + probe mixture, the DNA fragments with microsatellite repeat were captured using Dynabeads (Dynal, Oslo, Norway) under magnetic field. The amount of eluted DNA was increased by using the PCR enrichment to recover enriched DNA fragments using the super SNX forward primer with the appropriate PCR components under the conditions: 95 °C for 2 min; then, 25 cycles of 95 °C for 20 s, 60 °C for 20 s, 72 °C for 1.5 min; then 72 °C for 30 min. The enriched PCR product was directly cloned into TA vector (Invitrogen, U.S.). Plasmids were isolated and colony PCR was performed. The plasmids containing insert sizes of above 500 bp were selected and diluted to 100 ng/μL, and sequenced using automated sequencer (ABI prism 3700). The sequences were screened for the presence of microsatellites using the software MICAS (www.cdfd.org.in/micas/) after removal of redundant sequences. Primers were designed from the flanking region of the repeats of the non-redundant sequences using Primer3 software [9].

### 3.3. Detection of Polymorphism and Data Analysis

DNA was isolated individually from 10 female and 10 male adults from the iso-female families each of GMB1, GMB4 and GMB4M by Hot Shot protocol [20]. These DNA samples were used as templates directly for PCR with the optimized primers and PCR conditions. Amplified product was visualized on 10% PAGE stained with ethidium bromide, which resulted in the identification of 15 polymorphic markers. Of the 15 polymorphic markers, 10 markers were labeled with FAM fluorescent dye and genotyped in 3730 DNA Analyzer with HiDi formamide and GeneScan_TM_- 500LIZ^®^Size Standard (Applied Biosystems, U.S.). The results were analyzed with GeneMapper v4.0 software (Applied Biosystems, U.S.) to calculate the allele size and number of alleles. Genetic analysis was performed using Arlequin 3.1 [21], Genepop v4.0 [22] and Cervus v2.0 [11].

### 3.4. Inheritance of SSR Markers

Three markers (Oosat55, Oosat59 and Oosat43) were selected based on the observed polymorphism between males and females. These markers were selected for inheritance study in the iso-female families of GMB4M. Pedigreed crosses were made to obtain F_1_ and F_2_ progeny while insects after mating, were preserved, for genotyping with these markers. At least two parental pairs were used to generate F_1_ females that produced male and female progeny.

## 4. Conclusions

In conclusion, we report, for the first time, the development of 15 polymorphic microsatellite markers that can be used for efficient genetic studies, for example linkage analysis, and construction of molecular linkage maps. We also discovered markers that have sex linked inheritance in the gall midge. These markers are currently being screened, in a mapping population, to ascertain linkage with virulence alleles in the insect. This study could pave the way for identification of virulence genes in the insect. Further, these markers will be a good tool for developing strategies for the management of the rice gall midge.

## Supplementary Material

**Supplementary Table 1:** Microsatellite loci and primer sequences for the 75 SSR markers found to be monomorphic in three biotypes of the Asian rice gall midge, *Orseolia oryzae* (Wood-Mason) (Diptera: Cecidomyiidae).

Accession Number (GeneBank)	Locus	Repeat Motif	Primer	Ann. temp.	MgCl_2_ conc.	Alleles Size
HM804497	**Oosat01**	(GA)_10_	F:TCATCAAAAGGCAATGAGAAA R:GAAGACAACACACCGCACAT	59 °C	1.5 mM	159
HM804498	**Oosat02**	(TTAAAAT)_2_ N….(ATTTTA)_4_	F:TGCACAAAAATAACGCAGGA R:ATAACCCAACCAAACCACGA	60 °C	2.0 mM	263
HM804500	**Oosat04**	(TC)_10_…(TC)_3_	F:TGCACAAAAATTGCGATTCTAC R:CCCATATTGGGCAGCATCT	59 °C	3.0 mM	340
HM804501	**Oosat05**	(CA)_6_	F:ATGATCATGCTGCTGTGCTC R:TGCGCTATTCTCCCCAGTAG	61 °C	3.0 mM	134
HM804502	**Oosat06**	(GT)_4_…(CG)_2_...(GT)_4_...(GT)_5_	F:ATCTCAATCTTGGCGCTGTT R:TGCGAGCAATGAAACAAAAG	59 °C	1.5 mM	157
HM804503	**Oosat07**	(GT)_27_	F:TGCAGAATTCGGCTTAGTGA R:GGGCAAATTCTCTGTCTCGT	50 °C	2.0 mM	124
HM804504	**Oosat08**	(CA)_8_	F:GGCTTACTGCATCAGACTCTTTT R:AGCAGAATCGCTCTTTACGG	52 °C	2.0 mM	109
HM804505	**Oosat09**	(GT)_8_...(TG)_2_(CT)_2_	F:CGAATTATATGATCGCGGAAA R:AAAATGTGTTTTGGGTCGAGA	58 °C	1.5 mM	152
HM804506	**Oosat10**	(CA)_8_…(CA)_6_….(CA)_5_	F:TGACGTCAATAGCGACAACG R:GGCTTAGTGAGTGAGTGTGTGAG	56 °C	2.0 mM	164
HM804507	**Oosat11**	(GA)_5_	F:TCTCACAGTCGCATGTTATC R:ATGCTGAGAGCATCTGAAAT	56 °C	3.0 mM	110
HM804508	**Oosat12**	(TC)_9_	F:TATGCAAAGTGCGCGATATT R:TGTGCAGCCATTTACTGTGC	56 °C	2.0 mM	361
HM804509	**Oosat13**	(GA)_23_	F:GGGAAACGATGATGATAATG R:TTCAGTTGGTGAGTATTCATGT	52 °C	1.5 mM	145
HM804510	**Oosat14**	(TA)_3_...(GT)_4_...(GT)_2_	F:GTTCAGGCACCATGATATTT R:GTAGATTTTTCTTCGCAAGG	54 °C	1.5 mM	284
HM804511	**Oosat15**	(AG)_13_...(AG)_8_	F:TAATAAAAAGGGCTGTCTGC R:GCATACAGACAGAAAAATCAA	52 °C	3.0 mM	114
HM804513	**Oosat17**	(TA)_6_	F:CGAAATCGAACACAAACTTC R:GTTGAGCAGTTCAGGAGATT	55 °C	1.5 mM	115
HM804514	**Oosat18**	(CAA)_8_	F:GCCAGATAATGAAGCCGAGA R:GCCGAAATGCATTAATGGTC	52 °C	1.5 mM	255
HM804515	**Oosat19**	(CG)_4_(GC)_3_	F:AGGTGGAAGTGTTCGACGAG R:GAAGAACTCACGGTCGATGG	57 °C	2.5 mM	218
HM804516	**Oosat20**	(CA)_27_	F:TTTCGAAACGAAATCGAAAT R:GCTAGCAGAGTGGATGAGCA	52 °C	2.0 mM	111
HM804518	**Oosat22**	(GT)_13_	F:TTTGGGCCATGTATGAGAGC R:TGGAAGACTGAAGGGAAGACA	55 °C	2.0 mM	234
HM804519	**Oosat23**	(CA)_66_	F:TTTGGGCCATGTATGAGAGC R:TGGAAGACTGAAGGGAAGACA	59 °C	2.0 mM	206
HM804521	**Oosat25**	(GA)_6_…(GA)_3_	F:GTTGGTATCTGGTCGAGACG R:ACGCGCTTACCTGTTCAAAT	53 °C	2.5 mM	131
HM804523	**Oosat27**	(GA)_14_	F:TCGAAAATCAGCTGAACGAA R:AACTCTTACACCCACACATATTCA	52 °C	3.0 mM	129
HM804524	**Oosat28**	(GTT)_3_ ATT (GTT)_3_	F:CGCCTTTTTGCAAATTCTCT R:TGGATATTTGGTGTAAGGCAGA	51 °C	3.0 mM	115
HM804525	**Oosat29**	(ATT)_3_(ATG)_3_…(CAT)_2_	F:CGTTCATGTGAATTGGTTGG R:TGTATACGAAGGTGGCGATG	51 °C	3.0 mM	137
HM804526	**Oosat30**	(GTT)_7_	F:GCCGAAATGCATTAATGGTC R:TTCGATTATGGCATGGTTCA	50 °C	2.5 mM	140
HM804527	**Oosat31**	(TCCGTT)_2_…(GAATT)_2_ (TCCGTT)_2_..…(TCCGTT)_2_	F:ATCCGCGATCAATTATTCTG R:TCCAAGTCCGTGAAATCAAA	48 °C	3.0 mM	292
HM804528	**Oosat32**	(GA)_9_	F:CATGACACCATCCGATGAAT R:CACATAAACAACCAGGCACAA	50 °C	1.5 mM	105
HM804529	**Oosat33**	(AC)_6_..(AC)_6_..(AC)_3_..(AC)_7_.. (AC)_3_..(AC)_4_	F:CGACACACACGAAACACACA R:TTTCGGGCACCACTTTACTC	55 °C	2.5 mM	233
HM804530	**Oosat34**	(CAA)_12_	F:TGAGGCAGAATGAAAGAGCA R:CCATGGCACACGATAACAAT	51 °C	1.5 mM	156
HM804533	**Oosat37**	(GT)_10_	F:TTCGACCGACTGACTGAGTG R:GAGACGTCGGTCGTGATTTT	53 °C	1.5 mM	137
HM804534	**Oosat38**	(CTT)_6_	F:AACGGTTATAGAGTCGCGATG R:CGTGTGTTTCCTCACTAGAATCG	53 °C	1.5 mM	106
HM804535	**Oosat39**	(GT)_3_...(TG)_5_	F:AACTGGCCACGGTCATTATC R:AATACGTCGACGGAAGAACG	53 °C	1.5 mM	121
HM804536	**Oosat40**	(GT)(AT)(GT)_4_	F:GACCCAATCCACTTTGATCCT R:TGTCATCTAAAAGTATGTGCAACTGA	55 °C	1.5 mM	156
HM804537	**Oosat41**	(CT)_13_	F:TCGTTGGAATAGCACATTCG R:TGACGTGTCTATGCCATGTG	54 °C	1.5 mM	167
HM804538	**Oosat42**	(TAA)_7_(TAG)(TAA)_3_	F:GAGAGCAATTTTGATTCGACTTG R:GGGCCGAATGAAACAACTAC	54 °C	1.5 mM	150
HM804540	**Oosat44**	(GT)_17_	F:GAAAAGCCGTTCGTTGAATC R:TTTCCACCAAATAAGAAAACCA	50 °C	2.0 mM	193
HM804541	**Oosat45**	(GA)_8_…(GA)_6_	F:GAGTGAAAGAAGTCACGCACA R :GGCATCCACAGTCGAAGAT	50 °C	2.0 mM	145
HM804543	**Oosat47**	(GT)_21_	F:CGAAGTGAATGTTTAATGGTTT R:CCGGTTTGTATAATTGTGAA	56 °C	2.5 mM	128
HM804544	**Oosat48**	(GA)_14_…(GA)_8_	F:ACGCTGATCAAAAGAGTTCAG R:CCCTTGATAACAGAAAGTGAGAAC	52 °C	2.5 mM	116
HM804545	**Oosat49**	(CT)_10_GT(CT)_2_CA(GT)_2_AT (GT)_4_GA(GT)_20_	F:CAACGTCCCATAGTCTGCATT R:AGCGGCAGTGTTTTCTCTTC	55 °C	1.5 mM	168
HM804546	**Oosat50**	AG (AC)_2_ (TC)_2_ AC	F:TGAGATGATATGTTCCTTTTTGTC R:GCAGTTCCGAGATGTTTGTG	53 °C	1.5 mM	162
HM804547	**Oosat51**	(AT)_3_…(TA)_5_…(AT)_4_	F:GGTTTGACGGGCACTGTAT R:CGGCCACTGTATCTATAGGC	50 °C	2.0 mM	269
HM804548	**Oosat52**	(ACT)_3_(TGT)_4_	F:AACTTGGAATGAAGCGTTCG R:CGAGGTCTACCTCTACCCATAGAT	55 °C	1.5 mM	141
HM804549	**Oosat53**	(ATG)_4_…ATG	F:GAGTTGCTTTGAAACGATTGC R:ATCGTCGGATGAGTGTTTGA	54 °C	1.5 mM	110
HM804550	**Oosat54**	(GT)_8_	F:CTTGGCGTTTCGTTTATCTCA R:CCAATAAAGCAAGCACGTGTAA	54 °C	1.5 mM	149
HM804552	**Oosat56**	(GAAC)_4_…(GAAC)_4_ (GAAC)_4_	F:TGCGAACATTCAACGACCTA R:ACCACGCATACGTCAGGACT	56 °C	1.5 mM	138
HM804553	**Oosat57**	(TTG)_8_	F:CGATGTAGGCAACATTTTCG R:CCAATGAACATTCCCATCAA	52 °C	1.5 mM	100
HM804554	**Oosat58**	ACA (CAA)_6_ ACA	F:GTTCGGTCGGTGTCTTTTTC R:AAAGACCACACGCTGAAAGG	48 °C	2.0 mM	111
HM804556	**Oosat60**	(TA)_4_TC(TA)_2_TA TC (TA)_2_TT(AT)_6_	F:CCAGTGATTTGAGCATCGAG R:TCTTGGTTTTACGACCATTTCA	54 °C	1.5 mM	186
HM804557	**Oosat61**	(AGC)_2_N_5_(CAG)_2_ACA ..(GAC)_2_	F:GTCTGACTGGCATCACCAGA R:CGGCTATTTCCTGTCGGTAG	55 °C	1.5 mM	152
HM804558	**Oosat62**	(CA)_3_(CT)_2_(CA)_14_	F:GGAACCATTAAACACTCACTTCG R:GGAAATTCATGGTCCGAAAA	53 °C	1.5 mM	129
HM804559	**Oosat63**	(TG)_16_N_4_(CT)_6_	F:GAAGCACTGCAACAACCAAA R:TGTTCGCTCACACCGTTTAG	54 °C	1.5 mM	147
HM804560	**Oosat64**	(CA)_3_ AG (AC)_2_	F:TGAGACAGTTTTCGACTCCTTG R:TAGAGGGCTTTTTCGACTGC	54 °C	1.5 mM	131
HM804561	**Oosat65**	(GTT)_3_Nn(AT)CA(TC)_2_ (CA)_2_	F:TTCCTAGAATGTGGCGTTTG R:TTGAACGCAGGTTTAATTGC	50 °C	2.0 mM	194
HM804562	**Oosat66**	(ACA)_3_(TC)_2_CT(TC)_13_N_3_(CA)_14_	F:TGCATTTCCGACAGGTTTTA R:CACCTATCGTCTTAAAGGAAATGA	53 °C	1.5 mM	167
HM804563	**Oosat67**	(AT)_2_(TA)_3_	F:CTGTGCACACTTTGCCATTC R:ACTCCGTGTATGCGGAAAAG	55 °C	1.5 mM	202
HM804564	**Oosat68**	(TTTC)_3_	F:CGCATGAAATTTGGATCAGC R:ATCGTGCCAAAAGTGACTGA	54 °C	1.5 mM	101
HM804565	**Oosat69**	(TC)_7_CATA(CA)_3_CTCA	F:CCGGATAGATAGCCGTGTTT R:CTCACTTGGTGGGTGAGTACC	56 °C	2.0 mM	115
HM804566	**Oosat70**	(GA)_3_TA(GA)_3_Nn(GA)_4_	F:ATTTGGCCATGGCTATTTGA R:GCTGGGGGCTAATCTCTCTC	54 °C	1.5 mM	103
HM804567	**Oosat71**	(AT)_2_(AC)_3_ (CCA)_3_	F:GTGTGCGCACTTTACTGGTG R:TGTGGTGGATTTGCTTTTTG	55 °C	1.5 mM	199
HM804568	**Oosat72**	(CAT)N_2_ (CAT)_4_	F:TCGTCGGATGAGTGTTTGAT R:CAGAGGAGTTGCTTTGAAACG	54 °C	1.5 mM	114
HM804569	**Oosat73**	(TG)_12_	F:GGAAAACATGTCGGCAGAA R:TGCACATGGTGTTGTTGTTG	62 °C	2.0 mM	123
HM804570	**Oosat74**	(AG)_9_	F:TGAACATTGATACAGTGCGACA R:TGTGTCCGGGCCAATCTA	55 °C	1.5 mM	123
HM804571	**Oosat75**	(CT)_4_ N_3_ (TC)_7_ N_2_ (TC)_9_ N_2_ (CT)_7_	F:CAGTTTCGGTTCGTTTTTCA R:CTTGCCATCCATTCATCAGA	56 °C	2.0 mM	127
HM804572	**Oosat76**	(GTT)_5_	F:GGAAATTTTATTTCGGGAATTCAT R:ACCAAAGCTTTTCAACAACAG	52 °C	1.5 mM	190
HM804573	**Oosat77**	(GTC)_4_ GTT (GTC)_2_	F:CGAATTCAGCACGAACACTG R:ACGTTTTCGATCACCGTTTC	55 °C	1.5 mM	180
HM804576	**Oosat80**	(TC)_16_	F:TTGAAAAGTGAGGCTGATG R:TTAAACGTCCATCAAGTGAG	55 °C	1.5 mM	237
HM804577	**Oosat81**	(AG)_15_	F:TAAGCGATGTTGCTTGC R:CGATTTTGTCGTTGTGC	55 °C	1.5 mM	188
HM804578	**Oosat82**	(GT)_17_	F:AAATGAAAAGCCGTTCG R:TGCTAGCAGTTTCATTTCC	52 °C	1.5 mM	211
HM804580	**Oosat84**	(GT)_16_	F: CAATCGTTTCAGTTCCTTT R:CACCCAAAATTCAATCG	54 °C	1.5 mM	160
HM804581	**Oosat85**	(TC)_16_	F: CTAGCAGAATCACATTGA R:CAAATCATGCTCATAGTTCC	55 °C	1.5 mM	358
HM804582	**Oosat86**	(AG)_15_	F:TAAGCGATGTTGCTTGC R:GCGATTTGTCGTTGTGC	55 °C	1.5 mM	188
HM804583	**Oosat87**	(AC)_15_	F:TCCACCAAATACAGAAAACC R:AAATGAAAAGCCGTTCG	52 °C	1.5 mM	192
HM804585	**Oosat89**	(TG)_16_	F:GAAGCACTGCAACAACC R:GATCTGTTCGCTCACACC	55 °C	1.5 mM	151
HM804586	**Oosat90**	(TG)_16_	F: GAACATTATATTTTGAAAG R: AATGAAGCCTGAAGAAAGC	55 °C	1.5 mM	206

## Figures and Tables

**Table 1:** Microsatellite loci and primer sequences for the 15 SSR markers observed to be polymorphic in the three different biotypes of the Asian rice gall midge, *Orseolia oryzae* (Wood-Mason).

Accession Number (GeneBank)	Locus	Repeat	Primer	Ann. Temp (°C)	MgCl_2_ (mM)	Size Range	*n*	Biotype	He	Ho	PIC	HWE (P)
HM804499	**Oosat03**	(TG)_12_	*F:TTGATTGTCCCAAGGAGCAT	60	1.5	135–148 4	4	GMB1	0.583	0.350	0.475	0.0126
								GMB4	0.450	0.350	0.401	0.0020
			R:ATTCGCGTTGTGGATTGTTT					GMB4M	0.640	0.500	0.572	0.0550
HM804512	**Oosat16**	(TG)_15_ (GAGT)_6_	*F:TGTTCAGCTTGTTCAGC	55	1.5	153–162	8	GMB1	0.803	0.200	0.754	0.0000
								GMB4	0.560	0.100	0.478	0.0000
			R:CATTGGAACGAAATTAGTGG					GMB4M	0.810	0.500	0.770	0.0000
HM804517	**Oosat21**	(TA)_6_ (TG)_18_	F:CCGATTTCACTCGATGTTGTT	53	3.0	136–150	2	GMB1	0.518	0.000	0.365	0.0000
								GMB4	0.515	0.357	0.374	0.3150
			R:TTCTAACTTGAACTCCTCATTCG					GMB4M	0.508	0.286	0.374	0.1320
HM804520	**Oosat24**	(AC)_11_(CA)_5_	F:CCTCGGTCGCATCTCATATT	52	3.0	160–200	4	GMB1	0.634	1.000	0.501	0.0040
								GMB4	0.518	1.000	0.445	0.0000
			R:CCATTCAACAGATTTGGCGTA					GMB4M	0.645	1.000	0.548	0.0010
HM804522	**Oosat26**	(GT)_15_	*F:TGTCAGGTGGAACAGTAAATTG	53	3.0	214–236	9	GMB1	0.800	0.200	0.764	0.0000
								GMB4	0.750	0.450	0.686	0.0020
			R:GCCTGAAGAAAGCTGAATGAA					GMB4M	0.700	0.200	0.648	0.0000
HM804531	**Oosat35**	(CA)_11_ (GA) (GA)_2_	F:GCCCGTTGATTGCTTTGTAT	51	1.5	185–220	5	GMB1	0.796	0.928	0.704	0.0200
								GMB4	0.735	0.714	0.674	0.0000
			R:TATCGTTGTCGTCGTCTTCG					GMB4M	0.304	0.357	0.247	1.0000
HM804532	**Oosat36**	(GT)_14_	*F:CAGTTCCTTTTGTATATGCGTGAG	51	1.5	145–174	10	GMB1	0.780	0.800	0.738	0.0100
								GMB4	0.760	0.600	0.696	0.0070
			R:GCACCCAAAATTCAATCGTT					GMB4M	0.810	0.450	0.769	0.0001
HM804539	**Oosat43**	(CT)_9_	*F:TCGTTGGAATAGCACATTCG	54	1.5	165–188	7	GMB1	0.700	0.450	0.633	0.0200
								GMB4	0.380	0.400	0.305	1.0000
			R:TGACGTGTCTATGCCATGTG					GMB4M	0.620	0.500	0.557	0.0040
HM804542	**Oosat46**	(GA)_19_	F:AAATTGGCAGAGCGGAAGTA	44	2	185–250	3	GMB1	0.494	0.785	0.359	0.0360
								GMB4	0.648	1.000	0.553	0.0000
			R:TTTCACGGCCATCACATAAG					GMB4M	0.645	1.000	0.548	0.0010
HM804551	**Oosat55**	(CA)_2_ (CA)_20_	*F:CGTCGCCTTGTTGTAATATGTAAG	55	1.5	103–135	10	GMB1	0.780	0.500	0.744	0.0000
								GMB4	0.790	0.400	0.751	0.0000
			R:ACAGCCAATTGTGTTGCTTG					GMB4M	0.900	0.650	0.868	0.0000
HM804555	**Oosat59**	(CA)_20_	*F:CGTCGCCTTGTTTAATATG	55	1.5	78–107	11	GMB1	0.880	0.300	0.852	0.0000
								GMB4	0.580	0.150	0.534	0.0000
			R:CCAATTGTGTTGCTTGA					GMB4M	0.910	0.300	0.877	0.0000
HM804574	**Oosat78**	CAG (CAA)_2_ (CAG)_6_ CAA	F:CCCAGCTCTTCGAATTCTATTG	56	2	190–200	3	GMB1	0.476	0.000	0.305	0.0000
								GMB4	0.677	0.000	0.548	0.0000
			R:CCCGAATCATTTTGCATTGT					GMB4M	0.349	0.000	0.305	0.0000
HM804575	**Oosat79**	(TG)_11_	*F:CGCCCTAAAGAGTCGTGAAG	55	1.5	118–128	5	GMB1	0.350	0.400	0.329	1.0000
								GMB4	0.600	0.550	0.511	0.5940
			R:GAACCGGATGATTTGAATGG					GMB4M	0.680	0.600	0.611	0.8100
HM804579	**Oosat83**	(AG)_15_	*F:GCGAGTCAAAACACACG	55	1.5	105–120	9	GMB1	0.840	0.600	0.803	0.0000
								GMB4	0.710	0.500	0.646	0.0008
			R:ACACACACATATGCTCTTCC					GMB4M	0.790	0.250	0.742	0.0000
HM804584	**Oosat88**	(TC)_15_	*F:ACAGAAGGTAGAAGGAGAGC	55	1.5	184–192	6	GMB1	0.770	0.700	0.709	0.0200
								GMB4	0.710	0.650	0.665	0.2210
			R:AGTTGGCGATTGAGTGAG					GMB4M	0.760	0.600	0.693	0.0500

*n*: total number of alleles; He: Expected heterozygosity; Ho: Observed heterozygosity;

PIC: Polymorphic information content; HWE (*P*-value): Hardy Weinberg equilibrium

* FAM-labeled.

**Table 2:** Allele frequencies (most frequent alleles) of the polymorphic SSR markers amplified in different gall midge biotypes.

S/No.	Loci	Frequency of Most Frequent Alleles * (Size, bp) and Number of Alleles in Each Biotype					
		GMB1		GMB4		GMB4M	

		Frequency (Size)	No. of Alleles	Frequency (Size)	No. of Alleles	Frequency (Size)	No. of Alleles
**1**	**Oosat03**	0.475 (148)	4	0.674 (148)	4	0.525 (148)	4
**2**	**Oosat16**	0.350 (153)	6	0.600 (155)	5	0.325 (155)	8
**3**	**Oosat21**	0.600 (136)	2	0.525 (150)	2	0.525 (136)	2
**4**	**Oosat24**	0.500 (180)	4	0.475 (160,180)	4	0.500 (190)	3
**5**	**Oosat26**	0.350 (220)	9	0.350 (219)	6	0.500 (222)	6
**6**	**Oosat35**	0.375 (190)	5	0.375 (200)	4	0.825 (200)	2
**7**	**Oosat36**	0.350 (150)	8	0.350 (153)	5	0.375 (152)	10
**8**	**Oosat43**	0.425 (188)	7	0.750 (168)	2	0.550 (166)	5
**9**	**Oosat46**	0.625 (185)	2	0.500 (250)	3	0.500 (185)	3
**10**	**Oosat55**	0.400 (112)	8	0.400 (125)	9	0.175 (125)	10
**11**	**Oosat59**	0.200 (83,84)	11	0.625 (97)	6	0.150 (79,84)	11
**12**	**Oosat78**	0.750 (200)	2	0.500 (210)	3	0.750 (200)	2
**13**	**Oosat79**	0.800 (122)	5	0.500 (122)	3	0.425 (122)	5
**14**	**Oosat83**	0.250 (115,116)	9	0.450 (116)	5	0.350 (116)	6
**15**	**Oosat88**	0.325 (192)	5	0.475 (184)	6	0.300 (184)	4

* *N* = 60 (10 male and 10 female adults of the three biotype screened).

**Table 3:** F-Statistics and gene flow for all loci.

S/No.	Locus	FIS	FIT	FST	Nm
**1**	**Oosat03**	0.2666	0.3055	0.0531	4.4625
**2**	**Oosat16**	0.6238	0.6597	0.0955	2.3669
**3**	**Oosat21**	0.4585	0.4643	0.0106	23.3289
**4**	**Oosat24**	−0.7118	−0.3986	0.1830	1.1162
**5**	**Oosat26**	0.6147	0.6618	0.1222	1.7961
**6**	**Oosat35**	−0.1095	0.0625	0.1550	1.3624
**7**	**Oosat36**	0.1965	0.2851	0.1103	2.0168
**8**	**Oosat43**	0.1922	0.3466	0.1912	1.0578
**9**	**Oosat46**	−0.6058	−0.4808	0.0779	2.9611
**10**	**Oosat55**	0.3608	0.4178	0.0892	2.5526
**11**	**Oosat59**	0.6769	0.7164	0.1223	1.7948
**12**	**Oosat78**	1.0000	1.0000	0.1772	1.1610
**13**	**Oosat79**	0.0380	0.1296	0.0952	2.3753
**14**	**Oosat83**	0.4114	0.4348	0.0396	6.0628
**15**	**Oosat88**	0.1116	0.1551	0.0489	4.8598

FIS: fixation index (inter-individual); FST: fixation index (subpopulations); FIT: fixation index (total population).

Nm = Gene flow estimated from FST = 0.25(1 – FST)/FST.
